# Lessons from tissue-specific KO of CNP/GC-B system - everlasting challenge to clinical application

**DOI:** 10.1186/2050-6511-14-S1-O4

**Published:** 2013-08-29

**Authors:** Kazuwa Nakao, Koitiro Kuwahara, Hiroaki Yasoda, Goro Katuura, Kazuhiro Nakao, Kazumasa Nakao, Yui Yamasita

**Affiliations:** 1 Medical Innovation Center and Dept. of Med. and Clin. Sci., Kyoto Univ. Graduate School of Med., Kyoto, Japan

## 

Since the identification of the first natriuretic peptide, ANP, from the atrium of the heart, thirty years have passed and clinical applications have been achieved in ANP⋅BNP/GC-A system. In contrast with the distribution of the ANP⋅BNP/GC-A system as the endocrine system, the CNP/GC-B system is distributed as the paracrine/autocrine system.

In order to assess physiological rolls of the CNP/GC-B system, we have developed tissue –specific knockout mice using the Cre-Lox-P system. Here, we present results of both CNP knockout mice and GC-B knockout mice in the brain, bone and cardiovascular system and discuss the possible physiological and pathophysiological implications of the CNP/GC-B system.

